# Triazavirin—A Novel Effective Antiviral Drug

**DOI:** 10.3390/ijms232314537

**Published:** 2022-11-22

**Authors:** Oleg N. Chupakhin, Vladimir L. Rusinov, Mikhail V. Varaksin, Evgeny N. Ulomskiy, Konstantin V. Savateev, Ilya I. Butorin, Weijie Du, Zhiyong Sun, Valery N. Charushin

**Affiliations:** 1 Department of Organic and Biomolecular Chemistry, Institute of Chemical Engineering, Ural Federal University, 620002 Ekaterinburg, Russia; 2 Institute of Organic Synthesis, Ural Branch of the Russian Academy of Sciences, 620990 Ekaterinburg, Russia; 3 Department of Pharmacology, The Key Laboratory of Cardiovascular Research, Ministry of Education, College of Pharmacy, Harbin Medical University, Harbin 150086, China

**Keywords:** triazavirin, riamilovir, SARS-CoV-2, COVID-19, antiviral therapy

## Abstract

This review outlines the data of numerous studies relating to the broad-spectrum antiviral drug Triazavirin that was launched on the Russian pharmaceutical market in 2014 as an anti-influenza drug (the international non-patented name is Riamilovir). The range of antiviral activity of Triazavirin has been significantly expanded during recent years; in particular, it has been shown that Triazavirin exhibits activity against tick-borne encephalitis, Rift Valley fever, West Nile fever, and other infections of viral etiology. This drug has been approved for treatment of influenza and acute respiratory infections by the Russian Ministry of Health on the basis of comprehensive clinical trials involving over 450 patients. Triazavirin was found to be a highly effective and well-tolerated drug, allowing its over-the-counter sale. The recently published data on the use of Triazavirin in clinical practice for the treatment of patients with COVID-19 are discussed, with special attention paid to potential biological targets for this drug.

## 1. Introduction

The unprecedented coronavirus infection (designated as the SARS-CoV-2 or COVID-19 pandemic) that the world has been facing in recent years has affected over 630 million people worldwide and has already led to 6.6 million of deaths. Although a number of effective vaccines have been developed in a short time, oral therapeutics against SARS-CoV-2 are urgently needed to prevent more severe infection and to improve the quality of life for millions of patients.

Many research groups are involved now in the development of drugs for treatment or prevention of COVID-19. The time required to develop a new drug appears to be too long, which is unacceptable in the context of the current emergency. This is why great interest is now focused on the repurposing of approved antivirals to redirect them against COVID-19. A number of antivirals are now under investigation, predominantly those that are active against RNA viruses. The list of these drugs includes remdesivir, favipiravir, umifenovir, triazavirin, baloxavir, marboxil and others [1].

**Scheme 1:**
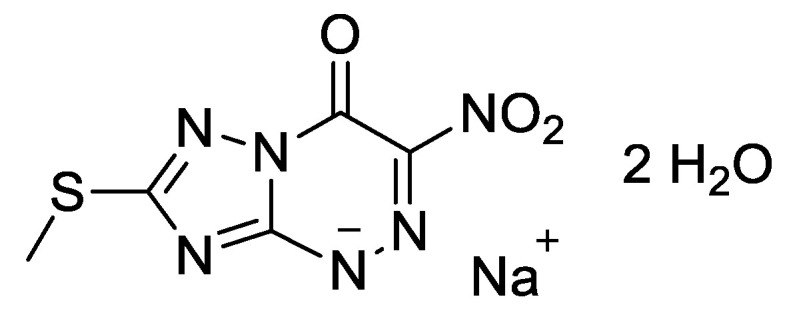
Structure of Triazavirin (Riamilovir).

Triazavirin (TZV), C_5_H_3_N_6_O_3_SNa 2H_2_O (sodium salt of 7-methylthio-3-nitro-6H-[1,2,4]triazolo[5,1-*c*][1,2,4]-triazin-4-one, dihydrate), (CAS Registry Number: 123606-06-4), also known as Riamilovir (international unpatented name), is an original antiviral drug of the azoloazine family [2], developed and registered in Russia [3] (Scheme 1).

This substance is effective against a wide range of influenza viruses, including H1N1, H3N2, H5N1, H5N2 and H9N2 strains [3,4,5,6,7]. It is worth noting that TZV is a drug with rather low toxicity. According to the results of acute toxicity tests, TZV is classified as a practically non-toxic drug [3,6]. In recent years, the spectrum of antiviral activity of TZV and its derivatives has been significantly expanded in accordance with data from the latest studies that have demonstrated its activity against parainfluenza, the Dengue virus, tick-borne encephalitis, Rift Valley fever, and the California encephalitis strains. The data concerning activity of TZV against the respiratory syncytial virus (strain Long), Crimean-Congo hemorrhagic fever, Rift Valley fever (strain Entebbe), West Nile fever and other viral infections have been patented in several countries [8,9,10,11,12,13,14,15,16,17].

The high efficacy and good tolerability of TZV in the therapy of influenza [18] and acute respiratory infections [19] were established in the course of clinical trials involving over 450 patients. Subsequent meta-analysis of these data allowed the Russian Ministry of Health to expand use of this drug with permission for over-the-counter sales.

Special attention will be paid to the data from clinical trials concerning treatment of COVID-19 patients with TZV [20,21,22,23,24,25,26,27]. A great deal of positive experience in this field has been obtained in a number of medical centers of the Russian Federation, including the Military Medical Academy named after S.M. Kirov (St. Petersburg), Polyclinic No. 3 of the Administration of the Russian President, the Research Institute of Pulmonology of the Russian Federal Medical and Biological Agency, the Federal Medical Biophysical Center named after A.I. Burnazyan, the clinical units of Krasnoyarsk, Samara and Ural State Medical Universities, as well as the City Clinical Hospital No. 14 in Ekaterinburg [20,21,22,23,24,25].

It is worth noting that TZV’s efficacy has been confirmed through the treatment of patients diagnosed by PCR test [20,21,22], and also as a preventive measure [24], as well as its use in combination therapy [23,25]. The good safety profile of Riamilovir (Triazavirin) with the absence of side-effects, has enabled recommendation of this drug as the first-line outpatient therapy for patients with COVID-19 based on the principle of multiple exposures [23].

This review considers synthetic approaches to TZV, its chemical transformations, the data on antiviral activity, as well as data on the interaction of TZV with viral proteins and other biotargets. The drug is referred to as either Triazavirin or Riamilovir, as cited in the original papers.

## 2. Approaches to Synthesis of Triazavirin and Chemical Properties of Nitroazoloazines

### 2.1. Synthetic Approaches

**Scheme 2:**
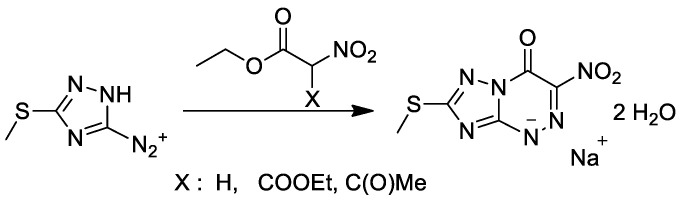
The key process in the synthesis of TZV.

Annelation of the 1,2,4-triazine ring fragment, containing the nitro group, to the 1,2,4-triazole moiety is the main synthetic method for preparation of Triazavirin. The key reactant for this process is 3-methylthio-1,2,4-triazolyl-5-diazonium salt obtained in situ from 3-methylthio-5-amino-1,2,4-triazole. α-Nitroesters, such as nitroacetate [2,10,11,28], nitromalonate [13,29], and nitroacetoacetate [29], act in this reaction as synthetic equivalents of the C-C building block, bearing the nitro group (Scheme 2).

**Scheme 3:**
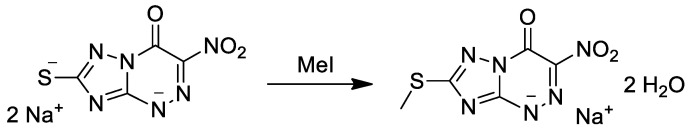
S-Alkylation as alternative synthetic route for obtaining TZV.

Alkylation of the disodium salt of 7-mercapto-3-nitro-1,2,4-triazolo [5,1-*c*]-1,2,4-triazin-4-one with methyl iodide is an alternative option for the synthesis of Triazavirin [29] (Scheme 3).

**Figure 1:**
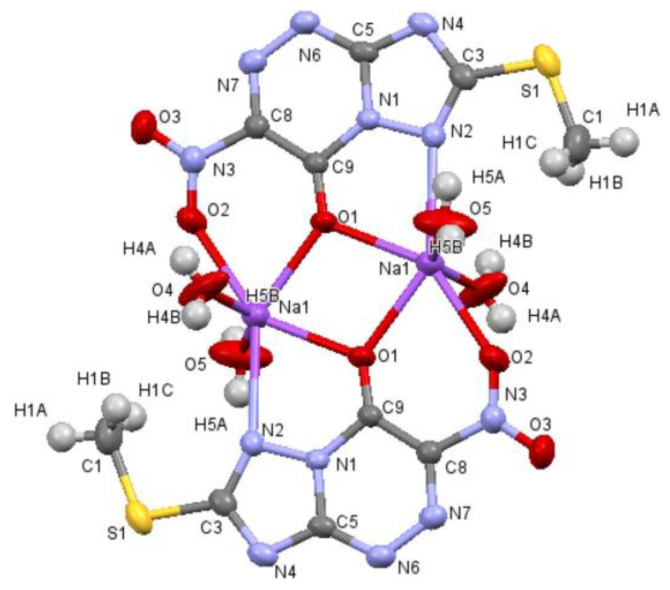
View of the dimeric structure, drawn with 50% probability displacement ellipsoids and showing the atom labeling. H-atoms are shown as small spheres of arbitrary radii. Reprinted/adapted with permission (license 5427551466931) from [30], 2018, Springer Nature.

It is worth mentioning that in a crystalline state, Triazavirin forms the dimeric structure with four molecules of water, which are coordinated with sodium cations, according to the X-ray crystallography data [30] (Figure 1).

**Scheme 4:**
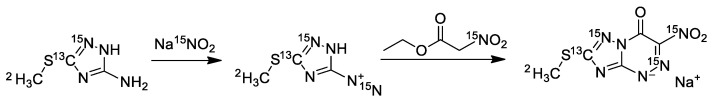
Synthesis of the selective isotope-labeled TZV.

Triazavirin with several isotopic labels, bearing 2H, 13C, and 15N atoms, was first synthesized according to Scheme 4 in order to study the metabolism of the drug [31,32].

### 2.2. Chemical Properties of Nitroazoloazines

**Scheme 5:**
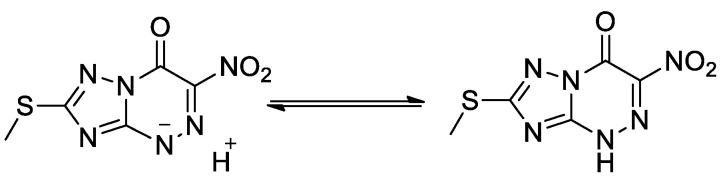
Acidic NH-form of TZV.

Chemical properties of Triazavirin provide a key basis to comprehend rational approaches for study of the bioavailability, mechanism of action, and biological functions of this drug. In this regard, it should be noted that one of the fundamental chemical properties of Triazavirin is NH-acidity (Scheme 5), which allows the sodium salt (dihydrate) to be obtained through the patented synthetic procedure [11].

**Scheme 6:**
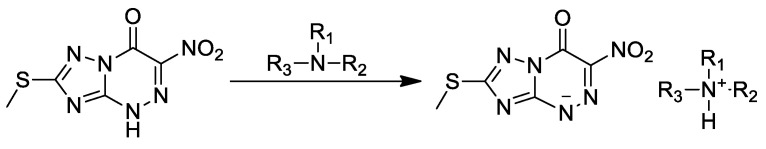
Formation of stable alkylammonium salts of TZV.

The dissociation constant for Triazavirin has not so far been reported, but the value pK_a_ = 1.1 for close structural analogues (for instance, 3-nitro-6H-[1,2,4]triazolo[5,1-*c*][1,2,4]-triazin-4-one) has been established [33]. The acidic character of Triazavirin enables one to obtain stable salts with a number of alkali metal cations, as well as with N-alkylammonium cations on treatment with amines [2,10] (Scheme 6).

**Scheme 7:**
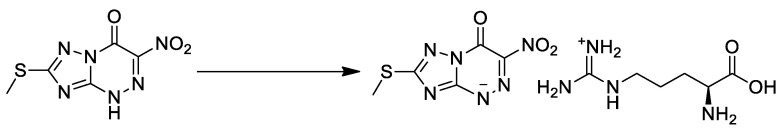
Synthesis of L-arginine salt of TZV.

The method was extended to obtain salts of Triazavirin with amino acids, in particular with L-arginine (Scheme 7).

**Scheme 8:**
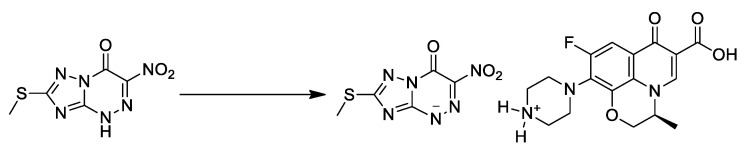
Ionic structure bearing Triazavirin and Fluoroquinolone moieties.

The latter ionic compound shows significant antiviral activity against influenza and the West Nile virus [34,35,36], while other ammonium salts proved also to exhibit antiviral effects [10]. Furthermore, the ionic structures bearing Triazavirin and Fluoroquinolone fragments have demonstrated a good level of antiviral activity against Venezuelan encephalitis, West Nile fever, Rift Valley fever, and the Dengue, in combination with a pronounced antimicrobial action against anthrax, tularemia and other bacterial infections [37,38] (Scheme 8).

**Scheme 9:**
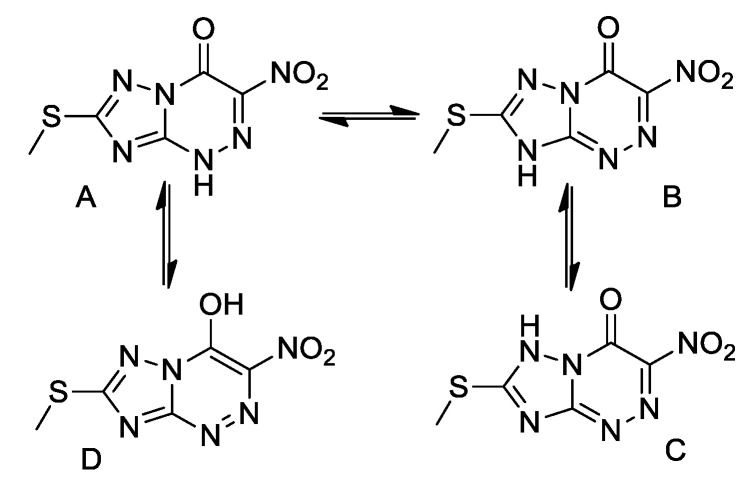
Tautomeric NH-forms of TZV.

Four tautomeric NH-forms A–D can be suggested for Triazavirin due to prototropic tautomerism, which is regarded as one of the key chemical properties [39,40] (Scheme 9). It has been shown that tautomer A is the major one, and tautomer B is minor, while tautomers C and D have not been detected [39].

**Scheme 10:**
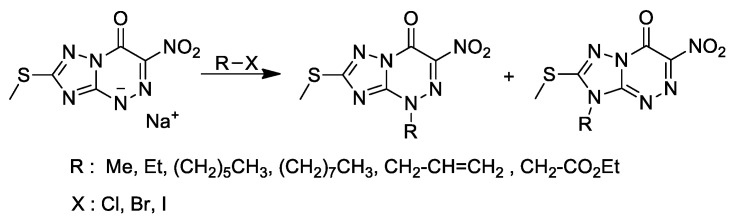
Unspecific N-alkylation of TZV.

The ability of Triazavirin and its analogues to form stable salts with alkali metal cations allows one to perform N-alkylation, which is of interest for several reasons. First of all, this structural modification of the azoloazine scaffold can afford novel derivatives with enhanced antiviral or antitumor activity. Secondly, the alkylation of Triazavirin is a process modeling the synthesis of nucleosides. A number and ratio of isomeric products derived from alkylation of Triazavirin studies enabled establishment of the spectral diagnostic features, thus allowing identification of the structure of more complicated nucleosides of the azoloazine family to be performed. It has been shown that alkylation of Triazavirin with alkyl halides leads to the formation of two isomers, and N4-alkyltriazolotriazine has been proved to be the major one [10,40], which is in agreement with the ratio of the corresponding tautomers A and B [39] (Scheme 10).

**Scheme 11:**
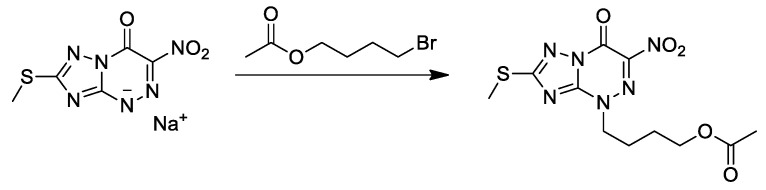
Regioselective alkylation of TZV with bromobutyl acetate.

The reaction of Triazavirin with bromobutyl acetate leads to the formation of the only N4-isomer [41], which can be regarded as a structural analogue of acyclic nucleosides (Scheme 11).

**Scheme 12:**
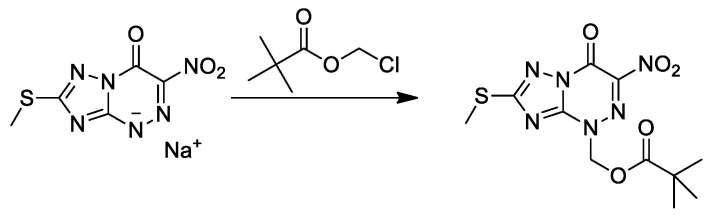
Regioselective alkylation of TZV with chloromethylpivalate.

In turn, alkylation of Triazavirin with chloromethylpivalate results in the formation of N4-product in 30% yield [42] (Scheme 12). The regioselectivity is possibly caused by steric hindrance preventing the formation of N3-isomer.

**Scheme 13:**
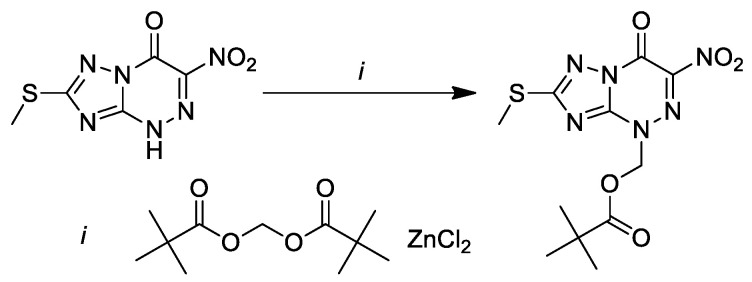
Efficient regioselective alkylation of NH-from of TZV with pivalic anhydride and catalytic amounts of ZnCl_2_.

The resulting alkylated product is considered to be an analogue of acyclic nucleosides due to structural similarity of the pivaloyloxymethyl fragment and the ribose moiety. Additionally, pivaloyloxymethyl plays the role of a protective group that undergoes cleavage under basic conditions. A more efficient synthetic method to obtain this N4-alkylated derivative is proposed later on the basis of the reaction of the Triazavirin NH-form with pivalic anhydride and paraformaldehyde in the presence of catalytic amounts of ZnCl_2_ at 140 °C (Scheme 13) [43].

**Scheme 14:**
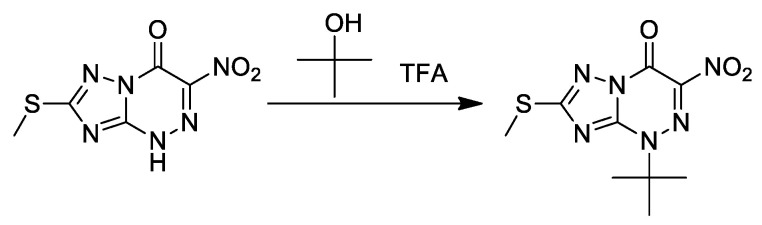
Regioselective alkylation of NH-form of TZV with *tert*-butanol in trifluoroacetic acid.

Furthermore, it has been found that alkylation of Triazavirin in the NH-form proceeds in the presence of Lewis or Brønsted acids. Thus, N4-*tert*-butyl derivative was obtained in excellent yield by reacting NH-Triazavirin with *tert*-butanol in trifluoroacetic acid at room temperature (Scheme 14) [43].

**Scheme 15:**
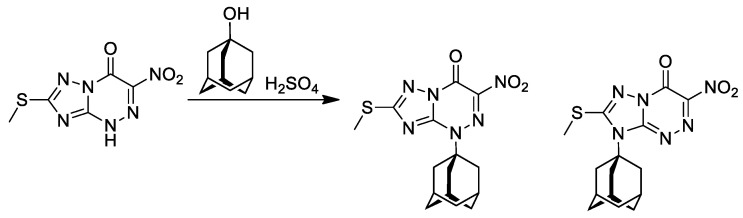
Unspecific alkylation of NH-form of TZV with adamantanol in sulfuric acid.

The reaction of triazolotriazine with adamantanol in sulfuric acid results in a mixture of isomers, while their ratio is determined by reaction conditions [44], indicating the reversibility of N-alkylation and the dependence of the reaction outcome on kinetically or thermodynamically controlled conditions (Scheme 15).

**Scheme 16:**
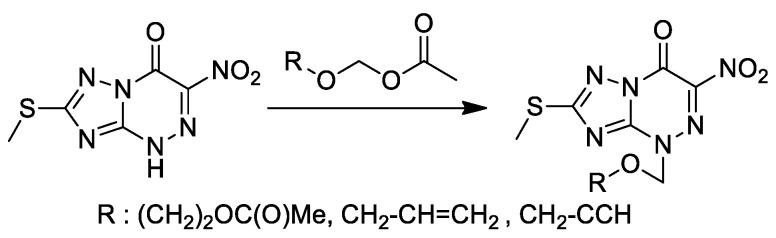
Regiospecific synthesis of non-natural nucleoside derivatives of TZV.

Additionally, the reversibility of the reaction and the intermolecular mechanism of the process were substantiated by studies with isotope labels. A series of non-natural nucleoside analogues have been obtained by exploiting the regiospecific reaction of NH-Triazavirin with alkylating agents on heating under neat conditions (Scheme 16) [41].

Thus, non-natural nucleosides exhibiting a remarkable level of antiviral activity have been obtained by alkylation of Triazavirin and its conjugated NH-form under different conditions.

**Scheme 17:**
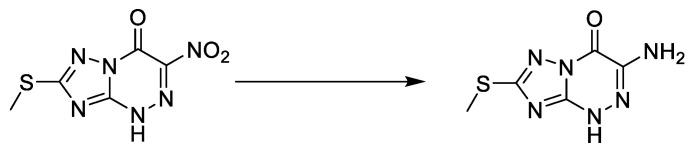
Reduction of the nitro group of NH-form of TZV.

In the course of studying potential metabolic pathways of 3-nitroazolo [5,1-*c*][1,2,4]-triazin-4-ones, it was found that one of the key and rapidly proceeding transformations is the reduction of the nitro group affording the corresponding amino derivatives (Scheme 17) [45]. In order to elucidate the role of these amino compounds, a synthetic approach for 3-aminoazoloazines needed to be developed. However, it was revealed that reduction of the nitro group on treatment with tin under acidic medium, or tin chloride, or catalytic hydrogenation leads to the targeted products in poor yields (<30%). At the same time, sodium dithionite proved to be a good reductive agent due to milder reaction conditions, easy isolation of the desired products, and good yields.

A pharmacokinetics study [6] revealed that the amino compound is a primary metabolite, which is easily accessible chemically through reduction of Triazavirin with sodium dithionite. Another possible metabolic pathway is oxidation of the alkylthio moiety, which takes place in the early stages of viral infections due to generation of rather reactive oxygen radical species and other active oxidants (O^•^, NO^•^, H_2_O_2_, ONOO^•^, HO^•^). It is known that influenza and other pathogenic viruses induce the nitric oxide synthase 2 (NOS2) to cause the formation of an excess of NO in tissues and peripheral blood, and conversion of the latter into the nitroperoxide anion (ONOO^•^) under oxidative conditions. A high concentration of the nitroperoxide anion leads to nitrosylation of proteins, their inactivation, and cellular disorders [46]. The typical oxidation process for methionine (SH-containing amino acid) can also be applied to transformation of the S-methyl group of Triazavirin, proceeding under oxidative stress in vivo along with conversion of the nitro group into unstable nitrosyl radical.

The processes mentioned above enable one to predict the sequence of metabolic transformations of Triazavirin in the human body; on the other hand, they provide an opportunity to simulate a covalent interaction of azolo [5,1-*c*][1,2,4]triazines with S- and N-fragments of the key proteins through the synthesis of the corresponding model compounds.

**Scheme 18:**
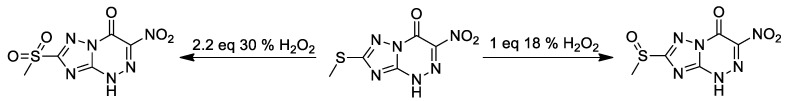
Synthetic routes of NH-TZV oxidation with hydrogen peroxide.

Studies on oxidation of Triazavirin and its structural analogues have revealed that the formation of the corresponding heterocyclic sulfoxides and sulfones takes place under appropriate reaction conditions. Indeed, treatment of S-alkylthio compounds with an equimolar amount of hydrogen peroxide in trifluoroacetic acid solution proved to result in the formation of the corresponding sulfoxides [43,47]. On the other hand, addition of 2.2 equivalents of 30% hydrogen peroxide solution to a suspension of 7-alkylthio-1,2,4-triazolo[5,1-*c*]triazines in trifluoroacetic acid at room temperature proved to afford sulfones in 62–71% yields (Scheme 18).

It was shown earlier that, when treated with nucleophiles of basic character, such as amines or alkalis, azolotriazinones are transformed into the corresponding salts, which are not able to undergo further nucleophilic attack. At the same time, N-alkyl substituted nitroazolotriazines are capable of reacting with nucleophiles through different pathways. Indeed, in the reaction of N-alkyl substituted azolotriazines with benzylamine substitution of the nitro group proceeds smoothly at room temperature, while “amino-denitration” substitution reactions by action of ammonia and other primary amines require one to raise the reaction temperatures up to 150 °C [48].

**Scheme 19:**
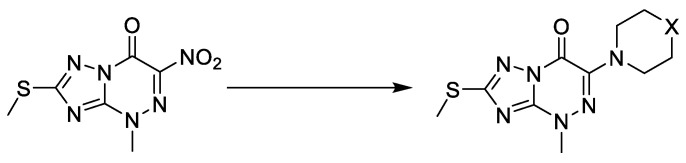
Substitution of the nitro group in N-alkyl substituted azolotriazines with N-nucleophiles.

Similar reaction conditions have been applied to cause substitution of the nitro group by action of cycloalkylimines on heating in DMF in the presence of three equivalents of the corresponding amino compound [49] (Scheme 19).

**Scheme 20:**
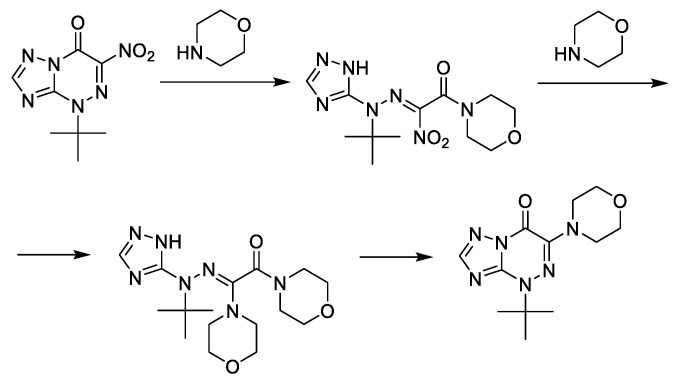
ANRORC process of the nitro group substitution in N-alkyl substituted nitroazolotriazines.

It has been found that the ANRORC mechanism is realized in many of these transformations, involving nucleophilic addition, ring opening of the triazine ring, substitution of the nitro group, and ring closure [49] (Scheme 20).

**Scheme 21:**
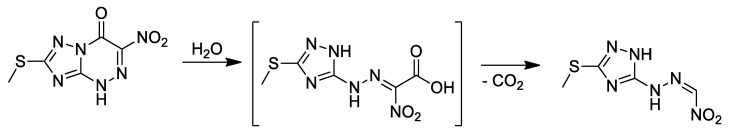
Opening of the triazine ring of NH-TZV provided by hydrolytic destruction.

Opening of the triazine ring has been observed in the course of hydrolytic destruction of Triazavirin and its structural analogues, and this process is considered to be a very plausible one in biological media [50] (Scheme 21).

**Scheme 22:**
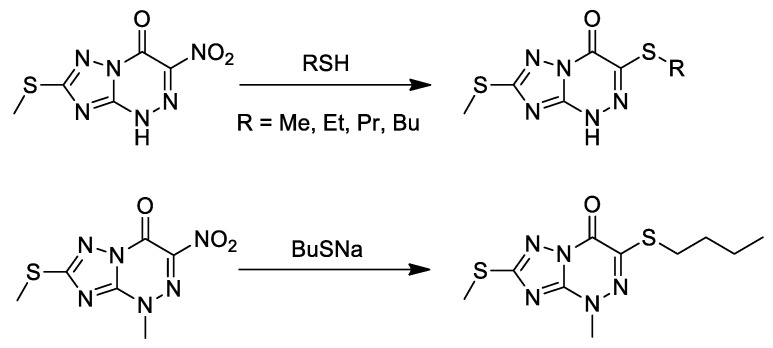
Substitution of nitro group of TZV derivatives with S-containing nucleophiles.

Heterocyclic thioethers have been synthesized through nucleophilic substitution of the nitro group in 7-methylthio-3-nitro-1,2,4-triazolo [5,1-*c*][1,2,4]triazine-4-one and its N-methyl derivative on treatment with thiols and thiolates [51] (Scheme 22).

**Scheme 23:**
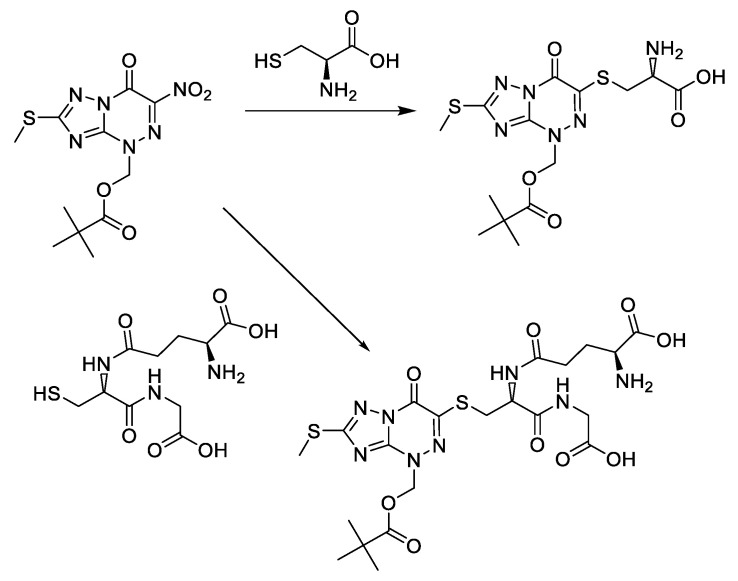
Nitro group substitution in TZV derivative with cysteine and glutathione.

It has been observed that the reaction of Triazavirin derivative with SH-containing amino acids and peptides proceeds according to the same mechanism. Thus, treatment of the pivaloyloxymethyl derivative of Triazavirin with cysteine or glutathione results in the formation of the corresponding products in which the nitro group is substituted with amino acid residues [48] (Scheme 23).

Transformations of Triazavirin derivatives by action of sulfur-containing amino acids and peptides can also be realized in vivo, and can be considered as chemical processes contributing to the mechanism of antiviral action of Triazavirin.

In summary, we have considered above the key chemical properties of Triazavirin, including:

- ability to form stable salts on treatment with alkali metals and amines;
- N-alkylation leading to acyclic nucleosides;
- reduction of the nitro group into the corresponding amino compounds;
- oxidation of SH and S-alkyl fragments;
- destruction of the 1,2,4-triazine ring;
- nucleophilic substitution of the nitro group.

It is worth noting that each of these properties can be responsible for the antiviral action of Triazavirin or its metabolites.

## 3. Pharmacological Properties of Triazavirin

### 3.1. Dosage Forms

A few dosage forms have been developed for Triazavirin, 7-methylthio-3-nitro-6H-[1,2,4]triazolo[5,1-*c*][1,2,4]-triazin-4-one, as active pharmaceutical ingredient. It can be administered as tablets and capsules, 250 mg [52,53,54] or solutions for injection [55]. A lipid composition of Triazavirin suitable for the preparation of liposomes coated with modified chitosan has also been developed. This combination of liposomes and a polysaccharide layer has been shown to increase colloidal stability for up to 3 months, and it demonstrates broad opportunities for surface modification [56,57]. Furthermore, an ultrasound method to produce an aerosol of Triazavirin has been developed [58,59]. This dosage form has been suggested for direct drug delivery to the lung, and it is supposed to be effective for the therapy of infections caused by viruses that have affinity to airway epithelial cells.

### 3.2. Antiviral Properties of Triazavirin

#### 3.2.1. Activity against Influenza Virus and Toxicology Profile

A number of research studies, including those of preclinical and clinical trials, have revealed that Triazavirin, which can be considered a guanine analogue, possesses pronounced activity against various strains of influenza virus, including H1N1, H3N2, H5N1, H5N2, H9N2, and H7N3 [3,4,5,60,61,62,63,64,65,66,67]. Triazavirin has successfully passed three phases of clinical trials, thus proving its clinical efficacy and safety in the treatment of adult patients with confirmed influenza diagnosis, as indicated in the State Register of Pharmaceutical Products for Medical Purposes of Russian Federation [3,7,9,60]. It is worth noting that Triazavirin has received considerable attention in recent years because of its activity against the new coronavirus infection, COVID-19 [20,21,22,23,24,25].

A low acute toxicity of Triazavirin was observed during in vivo tests: any signs of toxicity were absent when the drug was administered to outbred white mice weighing 10–12 g at the dose of 1000 mg/kg [8]. In turn, chronic toxicity studies have shown that Triazavirin at a dose of 200 mg/kg is well-tolerated when administered daily for 10 days [3,8]. The effect of Triazavirin on survival of mice with secondary bacterial influenza-associated pneumonia was also studied. Triazavirin at doses of 50 and 100 mg/kg per day provided a 67–75% survival rate in mice infected first with influenza virus A/CA/04/09 and then with *S. aureus*, while all 13 animals died in the negative control group [65].

It is considered that Triazavirin targets hemagglutinin, a specific viral protein, while the most plausible mechanism of action involves the inhibition of protein disulfide-isomerase; the latter enzyme is responsible for the formation and isomerization of disulfide bonds. This action of Triazavirin leads to disruption of the tertiary structure of hemagglutinin and the life cycle of the influenza virus [61].

Additional clinical trials were performed after Triazavirin was registered as an antiviral drug to specify its activity and safety. Thus, it was shown that Triazavirin (30 people, 750 mg/day) is comparable to Oseltamivir (29 people, 150 mg/day) in clinical efficacy for the treatment of patients with confirmed influenza diagnosis [66]. Another comparison, of Triazavirin (82 patients, 750 mg/day for 5 days) with Tamiflu (45 patients, 150 mg/day for 5 days), revealed that the times to recovery and the disappearance of fever, headache, and myalgia were shorter in the patience group administered with Triazavirin [63,64,66,67].

A meta-analysis of the data from randomized clinical trials on 471 patients with a confirmed influenza diagnosis (laboratory-confirmed presence of influenza virus antigens) has been performed according to PRISMA principles [18]. This analysis showed that administration of Riamilovir significantly affected the severity of clinical symptoms for patients with influenza and that this drug can be used in the initial therapy for adult patients with this diagnosis. Treatment with Triazavirin prevents the re-release of influenza virus RNA, as well as the risk of a severe course of the disease and duration of catarrhal symptoms, intoxication, and fever. In addition, it was confirmed that therapy with Triazavirin had statistically significant advantages, as indicated by a number of parameters, compared to both the placebo group and the Tamiflu (Oseltamivir) group [18].

#### 3.2.2. Triazavirin for Treatment of Acute Respiratory Virus Infections (ARVI) in Adults

A randomized, double-blind, placebo-controlled phase II clinical trial with 165 patients divided into 3 equal groups was performed under the aegis of several Russian research institutes in 2018–2019 to evaluate the efficacy, safety, tolerability, and optimal dose of Riamilovir (Triazavirin) for patients with ARVI. It was shown that Riamilovir (Triazavirin) was superior, relative to the placebo, in terms of time to persistent improvement of clinical symptoms and in time to normalization of body temperature. It was also found that use of the drug at doses of 500 mg/day and 750 mg/day proved to be comparable to the placebo in terms of safety and tolerability [68]. These results were confirmed later by a randomized, double-blind, placebo-controlled phase III clinical trial with 270 patients, showing that Riamilovir (Triazavirin) was superior to the placebo in terms of efficacy, while no side effects were established [69]. Additionally, intoxication and catarrhal respiratory syndrome were eliminated by the third day after administration of Triazavirin, while these symptoms remained in 55.8% of patients in the placebo group [69]. Thus, the administration of Riamilovir (Triazavirin) for ARVI treatment leads to rapid elimination of febrile intoxication and catarrhal respiratory syndromes and significantly reduces the risk of complications [5,70,71]. Furthermore, Triazavirin demonstrated its efficacy and safety in patients with ARVI or influenza with unfavorable somatic pathology (bronchial asthma, obesity, diabetes, etc.) [5,70].

Meta-analysis also confirmed significant correlations between the use of Triazavirin in both doses (100 mg five times daily or 250 mg three times daily) and the chance of achieving a sustained improvement in clinical symptoms by the fifth day of therapy (two studies, n = 435; pooled subgroup estimate of Riamilovir (Triazavirin) over placebo; OR [95% CI] = 1.76 [1492.09] (Z = 6.5; *p* < 0.00001)).The studies demonstrated that the use of Triazavirin is effective in both the initial and later stages of the disease, and, therefore, this drug is suitable for the initial therapy of adult patients with respiratory diseases of viral etiology [19].

#### 3.2.3. Activity against Tick-Borne Encephalitis Virus

Triazavirin was proved to be effective against tick-borne encephalitis virus in the course of research studies on embryonic porcine kidney epithelial inoculated line (SPEV). It was shown that administration of TZV suppresses reproduction of the virus in a broad concentration range and results in a decrease in the level of virus accumulation of 2.3 lg with 99.5% inhibition coefficient observed at the highest tolerated concentration of the drug [72].

Evaluation of Triazavirin in in vivo experiments against tick-borne encephalitis in white mice showed that the drug protected 55% of animals at a daily dose of 400 mg/kg according to the preventive scheme of administration (5, 2, and 1 day before infection). The same coefficient (55%) was observed for the emergency preventive scheme of administration (1, 24, and 48 h after infection) [73]. The protective effect of the therapeutic regimen of Triazavirin administration (24 h after infection and further daily for 4 days) proved to be 45% [74].

A study carried out in 2018 revealed that Triazavirin might be used in the complex treatment of tick-borne encephalitis in adults. Incorporation of Triazavirin into the treatment regimen of febrile forms of viral tick-borne encephalitis was accompanied by faster elimination of all clinical symptoms, including fever, intoxication syndrome, and catarrhal symptoms relative to the control [75].

#### 3.2.4. Activity against SARS-CoV-2

**Figure 2:**
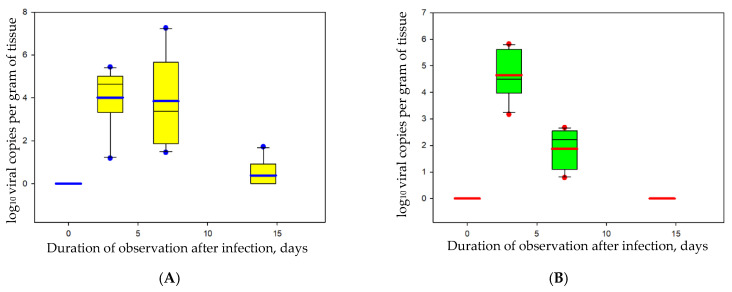
SARS-CoV-2 replication rate in the lungs of control (**A**) and Riamilovir (**B**) groups: (**A**) saline, 20 mL/kg, intraperitoneal, daily from 3 to 7 days after infection; (**B**) Riamilovir, 20 mg/kg, intraperitoneal, daily from 3 to 7 days after infection. Reprinted/adapted with permission (CC BY 3.0) from Ref. [76]. 2021, Chepur S.V. et al.

Data on Riamilovir (Triazavirin) effects on SARS-CoV-2 replication in lungs and dynamics of pathomorphological changes in internal tissues of Syrian hamsters have recently been published [76]. Animals in the studied group received the drug intraperitoneally every day at a dose of 20 mg/kg from day 3 to 7 after infection, while the positive control group received injections of saline in an equivalent volume, and intact animals were used as negative controls (Figure 2).

The effect of Riamilovir (Triazavirin) on viral replication in lung tissue was evaluated quantitatively using the polymerase chain reaction (PCR). It was revealed that Triazavirin reduced viral load by 3-fold at the end of therapy (7 days after infection), while no viral RNA was detected in the lungs of animals treated with Riamilovir. The authors calculated the characteristics of virus excretion in the positive control group and in animals treated with Riamilovir (Triazavirin) for 5 days at a dose of 20 mg/kg by exploiting the standard kinetic studies approaches. The results are summarized in Table 1.

The data obtained revealed that the rate of virus elimination from lung tissue was enhanced nearly 2-fold, while elimination half-life was shortened by approximately 1 day on treatment with Riamilovir (Triazavirin). Therefore, it was concluded that Triazavirin exhibits antiviral activity against SARS-CoV-2 virus in in vivo experiments [76].

The clinical efficacy of Riamilovir (Triazavirin) and the safety of the drug for treatment of patients with moderate COVID-19 were evaluated independently by several research teams in the Russian Federation (RF) [20,21,22,23,24,25,77] and The People’s Republic of China (PRC) [26,27].

Efficacy and safety of the drug in a dosage of 750 mg/day for 10 days were studied in a group of 214 patients with a moderate form of COVID-19. As a result, such clinical effects as relief of fever and laboratory signs of systemic inflammation were revealed [20]. Another research study involved evaluation of the efficacy of Riamilovir (Triazavirin) in the treatment of patients with moderate COVID-19 (29 patients, 1250 mg daily for 5 days) in comparison with a combination of Ribavirin and Umifenovir (30 patients, 800 mg daily for 5 days). Riamilovir was shown to reduce the level of nonspecific inflammatory markers in the blood serum along with normal characteristics of liver enzymes, in contrast to the group of patients receiving combined antiviral therapy. It is also worth noting that no side effects were observed in Riamilovir (Triazavirin) antiviral therapy [21].

The data from clinical trials involving two groups of patients with moderate COVID-19 diagnosis treated by either Riamilovir (Triazavirin) or combination of Umifenovir and Ribavirin are given in Table 2 and Table 3 [25].

Another group, of 120 patients with mild or moderate COVID-19 diagnosis, received Riamilovir (Triazavirin) in dosage of 750 mg/day for 7 days as monotherapy. As a result, the number (percentage) of patients with positive clinical dynamics after 3 days of monitoring was 21 (17.5%), after 10 days—117 (97.5%), while no significant side effects as indicated by clinical symptoms, abnormal laboratory parameters or EKG were observed [22].

A multicenter, randomized, placebo controlled, double-blind clinical trial was carried out in China on 52 patients with coronavirus infection, including severe cases [26,27]. It was shown that administration of Riamilovir (Triazavirin) in a dosage of 250 mg/day for 7 days provided the following changes:

- shortens the duration of major clinical symptoms, such as fever, and reduces the frequency of complications;
- improves patients’ responses to inflammation and hypercoagulation, reduces reliance on glucocorticoids, anticoagulants, and oxygen inhalation;
- results in a higher rate of recovery of abnormal serum bilirubin, indirect bilirubin, total protein, albumin, and uric acid levels;
- reduces use of electrolyte solutions and diuretics, resulting in less damage to liver and kidney function;
- the percentage of patients with clinical improvement in the Triazavirin group was nearly two times higher relative to the placebo group, and the average time for clinical improvement was 5 days shorter with administration of Triazavirin than with a placebo [26,27].

The prophylactic effect of Riamilovir (Triazavirin) (250 mg/day for 20 days) against COVID-19 infection has also been evaluated for persons having close contacts (family, work) with COVID-19 patients. High efficacy (97.35%) in foci of infection was identified through PCR test data, and no significant side effects were shown from use of the drug in the preventive scheme [24].

#### 3.2.5. Molecular Modeling Studies of Triazavirin against SARS-CoV-2

Currently, there are no biochemical studies dedicated to mechanisms of action of Triazavirin against SARS-CoV-2. 

According to virtual screening of the known antiviral drugs against SARS-CoV-2 on the basis of molecular docking data, Triazavirin is not capable of forming stable complexes with angiotensin-converting enzyme 2 (+13.77 kcal/mol), envelope protein (+11.68 kcal/mol), the spike protein (+6.05 kcal/mol), 3CL protease (+6.69 kcal/mol), membrane (+7.71 kcal/mol) and nucleocapsid proteins (+7.02 kcal/mol). Nevertheless, Yang and co-authors found the clinical use of Triazavirin to be rather promising due to a similarity of its binding pattern with that of 𝛽-d-N4-hydroxycytidine relative to ACE2, the spike protein and 3CL protease [77].

Later on, in a review article dedicated to drugs against SARS-CoV-2, the viral RNA-dependent RNA polymerase (RdRp) was suggested as the principal target for nucleoside/nucleotide analogues including Triazavirin, Ribavirin, Favipiravir, Molnupiravir and some other antiviral drugs [78]. So, one can assume a mechanism of action for Triazavirin by direct interaction with RdRp, or after its metabolism, that is similar to some active forms such as favipiravir [79], molnupiravir [80] and ribavirin (in silico) [81] metabolites. 

The main research study on molecular modeling of drugs against SARS-CoV-2 is focused on elucidation of the binding mode of potential antiviral drugs to the main protease (3CLpro). For example, Shahab et al. showed [82] a high affinity of Triazavirin (−9.94 kcal/mol) to the active site of the main protease due to the formation of hydrogen bonds with Asn142 and electrostatic interactions with His172, Glu166, Gly138, and Phe140 residues. In contrast, other molecular modeling studies revealed significantly lower binding energies of Triazavirin to 3CLpro due to other types of non-covalent interactions (Table 4).

Thus, at the moment, the exact mechanism of action of Triazavirin against SARS-CoV-2 has not been established. Based on the theoretical data described above, penetration, membrane, and nucleocapsid proteins can be excluded from the pool of plausible targets. Recent docking and molecular dynamics studies on 3CLpro indicate that the latter enzyme hardly plays a key role in the mechanism of antiviral action of Triazavirin. The target for Triazavirin could likely be found among other proteins involved in SARS-CoV-2 replication; some of them have recently been reported [78].

## 4. Conclusions

A number of preclinical and clinical data confirm the effectiveness of Triazavirin against a broad spectrum of viruses, including SARS-CoV-2. Moreover, its alkylated derivatives and salts with other alkali metals and ammonium cations also demonstrate significant or good antiviral activity against influenza, West Nile fever, Rift Valley fever, the Dengue, and Venezuelan encephalitis. The efficacy of Triazavirin against SARS-CoV-2 has been demonstrated in preclinical [76] and several clinical trials [20,21,22,23,24,25], including a multicenter, randomized, placebo controlled, double-blind trial [27]. Nowadays, according to some other studies [19,68,69] performed, Riamilovir is included in the guideline for ARVI treatment by the Ministry of Health of Russian Federation for use in etiotropic therapy against ARVI [86].

The mechanism of action of Triazavirin is still controversial. No experimental biochemical studies on the activity of Triazavirin relating to SARS-CoV-2 or influenza target proteins have so far been published. On the other hand, some experimental data and in silico studies of Triazavirin against the virus demonstrate hemagglutinin as a possible key target [61], which does not explain its activity against other viruses. Otherwise, molecular modeling of Triazavirin against SARS-CoV-2 proteins suggests two possible targets: RNA-dependent RNA polymerase and 3C-like protease.

Based on in silico studies, as well as the clinical efficacy of the drug’s usage in treatment [20,21,22,23,26,27] and for prophylactic purposes [24,73,74], and the evident similarity between Riamilovir and guanine, it can be assumed that Triazavirin is able to interact with such conservative proteins as RNA-dependent RNA-polymerase (RdPp) by direct interaction, or after possible metabolic transformations to active forms, as indicated in biochemical [20,21,22,23,26,27] and in silico studies for other nucleoside/nucleotide analogues—favipiravir [79], molnupiravir [80], and ribavirin [81].

Thus, good antiviral activity against a broad spectrum of viruses, extremely low acute toxicity, and proposed mechanisms of action present Triazavirin as an effective and well-tolerated over-the-counter drug. Nevertheless, more biochemical studies need to be concluded to gain insight into the mechanism of action of Riamilovir.

## Figures and Tables

**Table 1:** Elimination rate and elimination half-life of virus from lung tissue in female Syrian hamsters treated with Riamilovir (Triazavirin), 20 mg/kg, intraperitoneal, daily from 3 to 7 days after infection with SARS-CoV-2 (4.25 × 104 TCID50/mL) in dosage 26 µL/animal, intranasal. Adapted/Translated from Russian to English under the CC BY 3.0 license from Chepur, S.V., 2021 [76].

Group	Elimination Rate Constant, *k_e_,* Copies/g × Days	Elimination Half-Life, τ_1/2_, Days
Control	0.31	2.21
Therapy with Riamilovir, 20 mg/kg, intraperitoneal, daily from 3 to 7 days after infection	0.57	1.22

**Table 2:** Some characteristics of patients involved in the clinical trials. Reprinted/adapted with permission (CC BY NC SA 4.0) from Ref. [25]. 2021, Consilium Medicum.

Parameter	Number of Patients n = 69	Riamilovir Group (Number of Patients n = 34)	Ribavirin + Umifenovir Group (Number of Patients n = 35)
Age, years (M ± SD)	36 ± 3	39 ± 3	32 ± 3
Male, (%)	55 (79.7)	25 (73.5)	30 (85.7)
Body temperature, °C (M ± SD)	38.0 ± 0.8	38.0 ± 0.6	37.9 ± 0.9
Leukocytes (×10^9^/L), (M ± SD)	7.28 ± 1.35	5.76 ± 0.85	8.81 ± 1.54
Lymphocytes (×10^9^/L), (M ± SD)	1.6 ± 0.04	1.3 ± 0.02	2.08 ± 0.01
Thrombocytes (×10^9^/L), (M ± SD)	224 ± 13.45	228.39 ± 15.01	219.61 ± 11.74
Aspartate transaminase (IU/L), (M ± SD)	29.26 ± 4.27	28.36 ± 3.12	30.21 ± 5.41
Alanine transaminase (IU/L), (M ± SD)	29.37 ± 3.24	32.19 ± 4.34	26.54 ± 2.14

**Table 3:** Effects of Riamilovir (Triazavirin) and Umifenovir+Ribavirin antiviral therapy on hospital discharge rate and PCR test data in nasopharyngeal and oropharyngeal mucosa. Adapted/Translated from Russian to English under the CC BY-NC-SA 4.0 license from Kasyanenko, K.V., 2021 [25].

Number of Patients	Discharged by the 14th Day of Hospitalization	Not Discharged by the 14th Day of Hospitalization	Negative PCR Test after 7 Days of Hospitalization	Positive PCR Test after 7 Days of Hospitalization
Riamilovir	21	13	26	8
Ribavirin + Umifenovir	11	24	10	25
Overall	32	37	36	33

**Table 4:** Results for Triazavirin docking to the main 3CL-protease of SARS-CoV-2.

	Docking Score, kcal/mol	Non-Covalent Interactions	References
1	−9.94	Gly138, Phe140, Asn142, Glu166, His172	[82]
2	−5.8	His41, Cys145, His163, Met165	[83]
3	−5.52	Not defined	[84]
4	−5.8	Not defined	[85]

## Data Availability

Not applicable.
